# TAIGET: A small-molecule target identification and annotation web server

**DOI:** 10.3389/fphar.2022.898519

**Published:** 2022-08-29

**Authors:** Xuxu Wei, Jiarui Yang, Simin Li, Boyuan Li, Mengzhen Chen, Yukang Lu, Xiang Wu, Zeyu Cheng, Xiaoyu Zhang, Zhao Chen, Chunxia Wang, Edwin Wang, Ruiqing Zheng, Xue Xu, Hongcai Shang

**Affiliations:** ^1^ Key Laboratory of Occupational Hazard Identification and Control, Wuhan University of Science and Technology, Wuhan, China; ^2^ Key Laboratory of Chinese Internal Medicine of MOE, Dongzhimen Hospital, Beijing University of Chinese Medicine, Beijing, China; ^3^ School of Computer Science and Engineering, Central South University, Changsha, China; ^4^ Cumming School of Medicine, University of Calgary, Calgary, AB, Canada

**Keywords:** target prediction, web server, target annotation, cancer, botanical drug

## Abstract

**Background:** Accurate target identification of small molecules and downstream target annotation are important in pharmaceutical research and drug development.

**Methods:** We present TAIGET, a friendly and easy to operate graphical web interface, which consists of a docking module based on AutoDock Vina and LeDock, a target screen module based on a Bayesian–Gaussian mixture model (BGMM), and a target annotation module derived from >14,000 cancer-related literature works.

**Results:** TAIGET produces binding poses by selecting ≤5 proteins at a time from the UniProt ID-PDB network and submitting ≤3 ligands at a time with the SMILES format. Once the identification process of binding poses is complete, TAIGET then screens potential targets based on the BGMM. In addition, three medical experts and 10 medical students curated associations among drugs, genes, gene regulation, cancer outcome phenotype, 2,170 cancer cell types, and 73 cancer types from the PubMed literature, with the aim to construct a target annotation module. A target-related PPI network can be visualized by an interactive interface.

**Conclusion:** This online tool significantly lowers the entry barrier of virtual identification of targets for users who are not experts in the technical aspects of virtual drug discovery. The web server is available free of charge at http://www.taiget.cn/.

## Key points

•  TAIGET is a graphical web interface to identify potential targets of small molecules, which consists of a docking module, a target screen module, and a target annotation module.
•  The target annotation module is constructed by text mining and manually curating >14,000 cancer-related literature works, which involves 73 cancer types and 2,170 cell types.
•  TAIGET supports docking service in a mini-batch mode.

## Instruction

A drug discovery process starts with identification of targets and clarification of mechanism of action of drugs, with the hope to win the battle of disease treatment (Vamathevan et al., 2019). Approaches for target identification in drug discovery include virtual and experimental screening. As one of the most widely used structure-based virtual screening approaches, molecular docking allows identifying the most likely target of a query ligand. There are many popular docking procedures, such as AutoDock, LeDock, Glide, GOLD, and DOCK (Lapillo et al., 2019; Shahid et al., 2021). To reduce scoring bias, Lee and Kim, (2020) constructed a web server for target prediction, by ranking scoring algorithms of GOLD, AutoDock Vina, and LeDock. To assist identification of putative targets for herbal ingredients, Zhang et al. (2019) used a reverse docking approach to predict ligand–target interactions. When Ma and Zou, (2021) developed an inverse docking procedure using the DOCK algorithm to support docking ligands against an ensemble of multiple protein structures.

However, the advantages of docking are balanced by a serious deficit: docking creates many false-positive events (Lyu et al., 2019). This is caused by relatively rough search algorithms, for example, Monte Carlo algorithm generates a random initial configuration of ligand in the active site consisting of a random conformation, translation, and rotation; tabu search algorithm made a number of small random changes to the current configuration of ligand and ranked them (Sulimov et al., 2019). To avoid the false-positive events, we previously developed a target filter algorithm based on a Bayesian–Gaussian mixture model (BGMM) (Wei et al., 2022). We clustered the interaction pairs between ligand atoms and protein fragments extracted from the crystal structures of ligand-binding proteins in the PDB (released from January 1995 to April 2021) and found that the potential targets should meet with ≥600 significant interaction pairs, and meanwhile, ≥0.8 ratio of them to all the interaction pairs (Wei et al., 2022). The advantage of our method was that we not only considered the major bonds between the ligand and protein, such as hydrogen bonds, salt bridges, hydrophobic contacts, halogen bonds, and pi-stacking (Shaikh et al., 2021), but also summarized all the atomic contacts between the ligand and protein by defining an atomic contact between one ligand atom and the first atom of the protein fragment with an interatomic distance ≤5 Å. We proposed that the diverse characteristics of atomic contacts could accurately screen the targets of small molecules.

In addition, target annotation is important for researchers to identify functional elements of targets and to get an insight into target-related proteins/genes and their functions (Xu et al., 2022; Zhang et al., 2022). There are two main ways to annotate targets. One is based on literature curation or experimental results. Several popular databases provided information about protein/gene interactions collected from literature or experimental results, for example, IntAct molecular interaction database, BioGRID, and Molecular INTeraction (MINT) database (Licata et al., 2012; Orchard et al., 2014; Chatr-Aryamontri et al., 2015). The protein interaction network analysis (PINA) platform integrated protein–protein interactions (PPIs) with RNA-seq transcriptomes and mass spectrometry-based proteomes (Du et al., 2021). Another method is based on machine learning or deep learning. Sun et al. applied stacked autoencoder (SAE) to study sequence-based PPI prediction with an average accuracy of 97.19% (Hashemifar et al., 2018). Chen et al. presented a residual recurrent convolutional neural network in the Siamese architecture for PPI prediction. Zeng et al. (2020) developed an end-to-end deep learning framework with combined local contextual and global sequence features to predict PPI. The aforementioned studies raised the following three questions: 1) Different drugs have different effects on protein/gene regulation, so whether some PPIs could be broken by specific drugs? 2) Are PPIs different in different cell lines? 3) Are PPIs different in different diseases?

**FIGURE 1:**
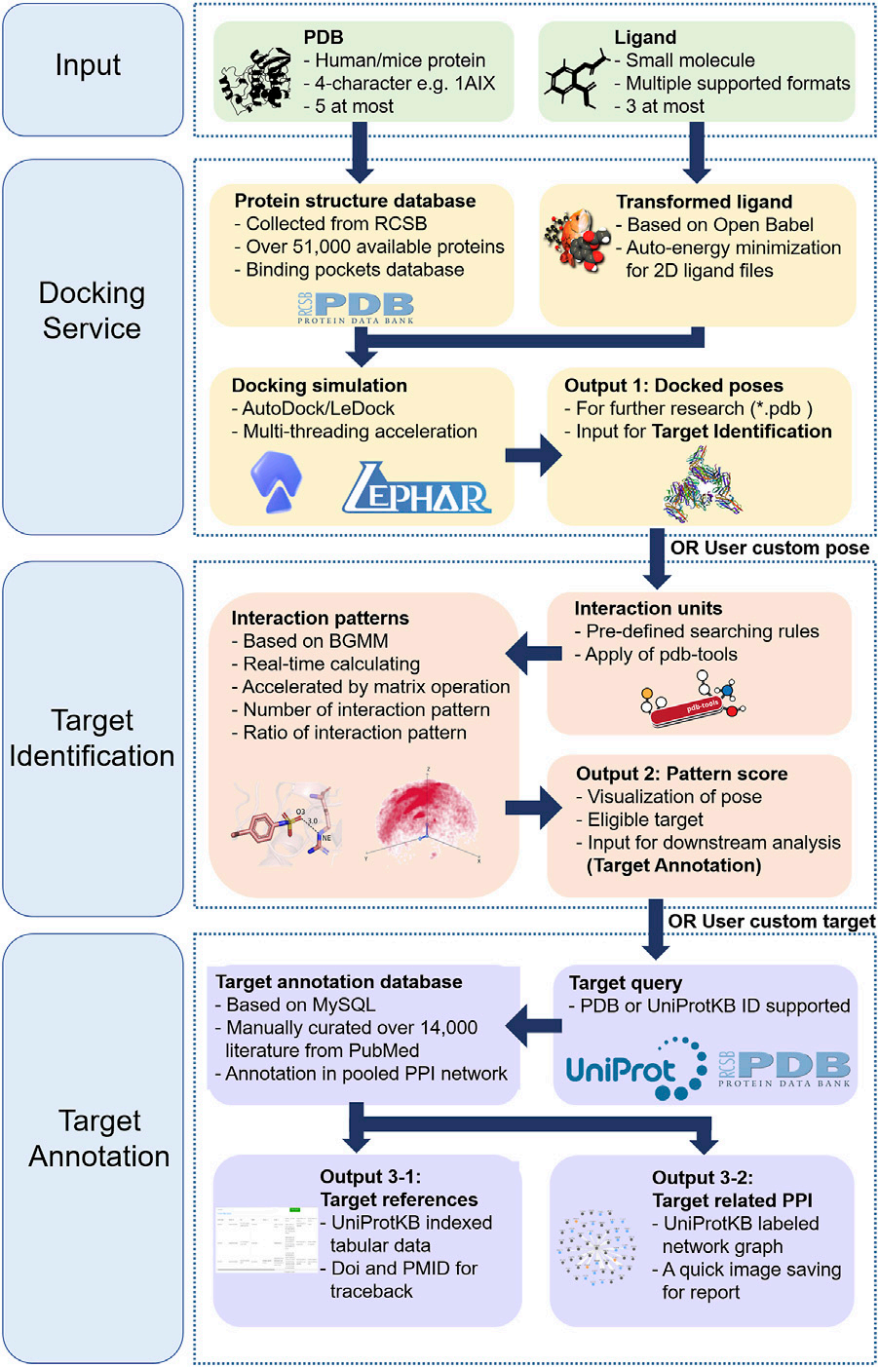
TAIGET workflow.

In this study, we developed TAIGET, a web server integrating target identification and annotation (Figure 1). We provide here the description of docking service, target screen, target annotation, and PPI analysis. User guide and examples of how to use TAIGET are further provided online.

## Implementation

We first collected 176,773 PDB files (January 1995–April 2021) from the Protein Data Bank (PDB). Only *Homo sapiens*/mouse protein–ligand binding files were maintained. After filtering of the PDB files based on our previous work (Wei et al., 2022), hydrogen atoms in proteins were removed by pdb-tools (Rodrigues et al., 2018) and were added again by Reduce. Size and position of binding pockets were calculated based on 3D-coordinates of ligands in the PDB files. A UniProt ID-PDB database was then constructed, consisting of 3D structures of 51,362 proteins named by four-character PDB IDs. The corresponding UniProt IDs of proteins were also involved in the UniProt ID-PDB database. Thus, users could provide UniProt IDs or four-character PDB IDs as protein inputs.

The details about the BGMM-based target filter algorithm could be found in our previous work (Wei et al., 2022). Briefly, protein–ligand binding structures in the PDB files were split into interaction pairs of ligand atoms and protein fragments (covalently linked three heavy atoms) with an interatomic distance of ≤5 Å. The interaction pairs were grouped into ligand atoms with the same SYBYL atom type surrounding the same protein fragment, which were further clustered via the BGMM. Gaussian distributions with ≥20 ligand atoms were identified as significant interaction patterns. Finally, the number of significant docked interaction pairs and the ratio of them to all the docked interaction pairs were defined as two important criteria to screen potential targets after docking.

To validate the importance of the aforementioned two features, we constructed a dataset involving 314 representative ligand–protein complexes from the PDB database in the previous work. Docking was conducted on the ligand and the corresponding protein involved in the 314 complex structures. For each docking case, root-mean square deviation (RMSD) was used to estimate structural similarity between the ligand poses and their corresponding crystal structure, respectively, and the ligand poses with the highest and lowest RMSD values were maintained. The process produced 1,252 binding poses. We further classified the binding poses into two groups with a threshold of RMSD of 2.5 Å. We proposed that the significant interaction patterns were reliable if the aforementioned two features were significantly higher in the group with RMSD ≤2.5 Å than in the group with RMSD >2.5 Å.

**FIGURE 2:**
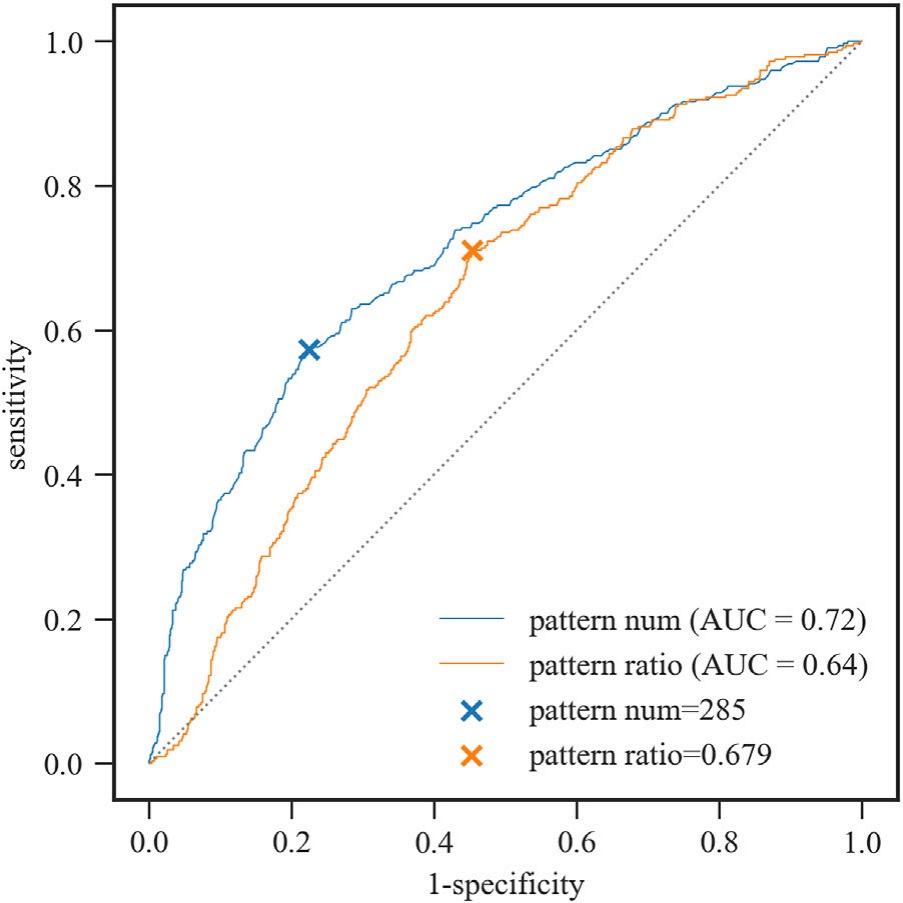
ROC curves for the group with RMSD ≤2.5 Å by the pattern number and pattern ratio, respectively. Pattern num: the number of significant docked interaction pairs. Pattern ratio: the ratio of significant docked interaction pairs to all the docked interaction pairs.

Here, we used ROC analysis of the two features to evaluate the classification accuracy. The Youden Index (YI) was used to obtain the optimal cut-off point (Figure 2). When the number of significant docked interaction pairs was equal to 285, the sensitivity at the maximal Youden Index was 0.573 for the group with RMSD ≤2.5 Å, while the specificity was 0.776. When the ratio of significant docked interaction pairs to all the docked interaction pairs was equal to 0.679, the sensitivity at the maximal Youden Index was 0.71 for the group with RMSD ≤2.5 Å, while the specificity was 0.547.

**FIGURE 3:**
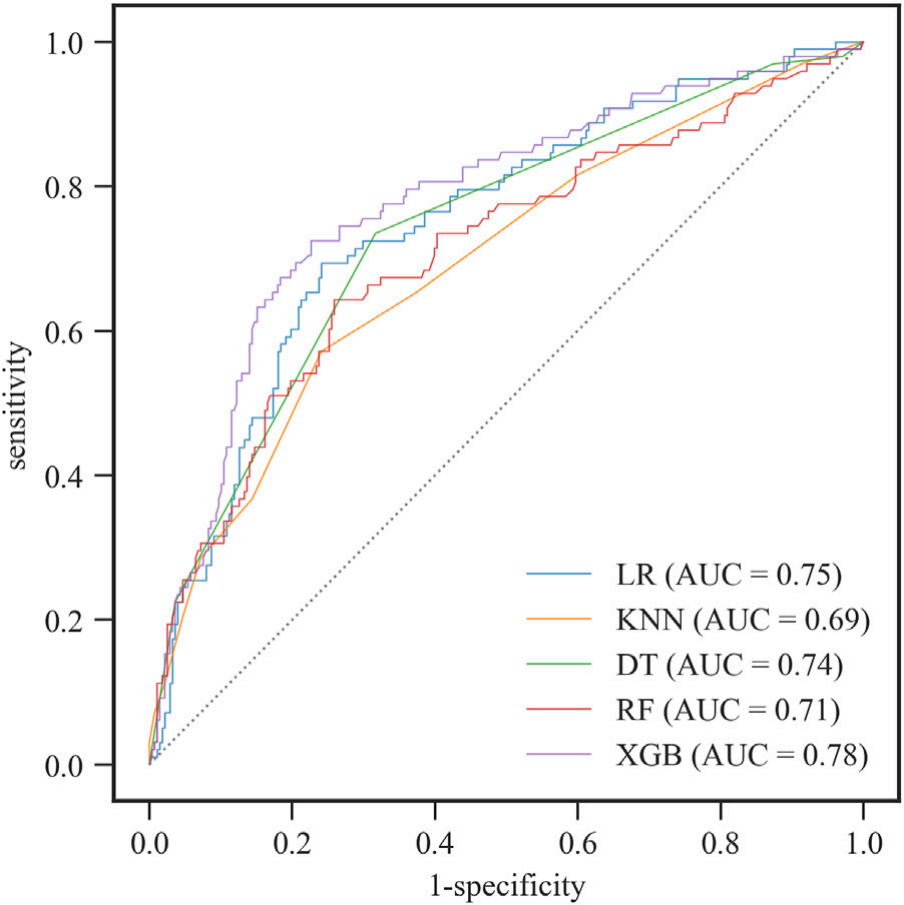
ROC curves for the group with RMSD ≤2.5 Å in the test set by machine learning models. LR: logistic regression, KNNs: k-nearest neighbors, DT: decision tree, RF: random forest, XGB: XGBoost.

Because the number of significant docked interaction pairs and the ratio of them to all the docked interaction pairs were two independent and non-linear correlated features, we further constructed machine learning models by using the two features to predict probability of the binding pose with RMSD ≤2.5 Å. We randomly divided the 1,252 binding poses into a training set (*n* = 876) and a test set (*n* = 376). Among logistic regression (LR), k-nearest neighbors (KNNs), decision tree (DT), random forest (RF), and XGBoost, XGBoost yielded the best AUC of 0.78 (Figure 3). As shown in Table 1, XGBoost in the test set showed 77% accuracy and 95% specificity. Thus, we used XGBoost as the classification model in TAIGET to predict the probability of obtaining a true-positive target.

**TABLE 1:** Model performance on the test set.

	AUC	Accuracy	Sensitivity	Specificity	PPV	NPV
LR	0.75	0.75	0.11	0.97	0.58	0.76
KNN	0.74	0.78	0.3	0.95	0.67	0.79
DT	0.74	0.77	0.22	0.96	0.69	0.78
RF	0.71	0.76	0.32	0.91	0.55	0.79
XGB	0.78	0.77	0.24	0.95	0.63	0.78

LR, logistic regression; KNNs, k-nearest neighbors; DT, decision tree; RF, random forest; XGB, XGBoost; PPV, positive predictive value; NPV, negative predictive value.

For target annotation, 55,299 cancer-related literature works ranging from 1995 to 2021 was first filtered by searching (cancer [Tile/Abstract]) AND (viability [Tile/Abstract]) OR (apoptosis [Tile/Abstract]) OR (invasion [Tile/Abstract]) OR (migration [Tile/Abstract]) AND (drug [Tile/Abstract]) in PubMed. Titles, abstracts, PMID, article types, and other related categories of the literature were downloaded. After removing reviews, meta-analyses, and clinical assays, information on PMID, species, drugs, genes, regulation, cancer cell lines, cancer subtypes, and cancer outcome was extracted from 14,394 literature studies. Three medical experts and 10 medical students then manually curated all the literature, especially in the following situations: 1) regularization for cell lines in the abstracts could not distinguish drug response in a specific cell line from many cell lines, for example, lanatoside C had an anti-proliferation effect on different human cancer cell lines (MKN-45, SGC-7901, HN4; MCF-7, and HepG2). MKN-45 cells treated with lanatoside C showed upregulation of cleaved caspase-9 and cleaved PARP and downregulation of Bcl-xl. Medical experts and students replaced “cancer cell lines (MKN-45, SGC-7901, HN4; MCF-7, and HepG2)” with “MKN-45 cell line.” 2) Regularization for cell lines in the abstracts could not distinguish the name of a specific cell line from the names of many cell lines, for example, breast cancer and melanoma cell lines were checked for the response to PTX by cytotoxic assay. Medical experts and students replaced “breast cancer and melanoma cell lines” with “breast cancer cell line” and “melanoma cell line.” Furthermore, gene information was standardized by protein ID in UniProt and protein-coding genes in GENCODE (Frankish et al., 2021), while cell line information was standardized by cancer cell lines in CCLE (Nusinow et al., 2020).

In addition, PPIs in AFFINOMICS and cancer and cardiac datasets in BioGRID (https://thebiogrid.org/) and IntAct (https://www.ebi.ac.uk/intact/) were combined, with the aim to construct target-related networks.

The TAIGET web server integrated, for the first time, a docking service module, a target screen module, and a target annotation module in a single GUI environment. Users have two ways to submit a protein or ligand: 1) selecting a PDB ID or a UniProt ID as a protein and 2) submitting a SMILES molecular formula or a ligand file prepared by users. Also, the users have two ways to run a job: 1) providing an input and sequentially running the job from step 1 to step 3 and 2) selecting a specific step among the three steps and providing the corresponding input to run a sub-job.

The following parameters are available in the “STEP1: Docking Service” module:

•  Input files: Users can provide protein by entering four-character PDB IDs or selecting UniProt IDs from our UniProt ID-PDB database. Users can provide ≤5 proteins at a time. In addition, users can provide ligands by entering SMILES molecular formulas or uploading ligand files in one of the following formats, that is, *.pdb, *.pdbqt, *.smi, *.sdf, and *.mol2. The SMILES molecular formulas of ligands can be transformed to 3D structures by Open Babel (O'Boyle et al., 2011) involved in TAIGET. Users can provide ≤3 ligands at a time.
•  Docking service: users can select AutoDock Vina (Trott and Olson, 2010) or LeDock (Wang et al., 2016) to run docking. AutoDock Vina and LeDock are the two popular academic docking tools with relatively high accuracy. However, although AutoDock Vina achieves a large docking success rate, the correlation between estimated and experimental binding free energy is low (R < 0.5) (Nguyen et al., 2020). The weakness of LeDock is its inability to calculate accurate binding energies. During the docking process in TAIGET, a progress bar will be shown.
•  Data download: when the docking is complete, users can download protein–ligand binding poses or click the “Go to step 2” button.
•  Run time: ∼10s is required for each pair of protein and ligand.

The following parameters are available in the “STEP2: Target Screen” module:

•  Input files: if users click the “Go to step 2” button, TAIGET starts the target screen immediately. Users can also provide a protein–ligand binding pose created by themselves in this step.
•  Target screen: when the job running is finished, a table, portraying protein–ligand poses, PDB names, ligand names, interaction patterns, interaction pattern ratios, and probability will be shown.
•  An interactive image: when users click a specific row of the table, a 3D protein–ligand binding structure will be shown on the left window. Users can rotate the image by the mouse.
•  Data download: users can download the target-related matrix and the 3D image.
•  Run time: ∼30s is required for the identification of interaction pairs and the pattern calculation for each protein–ligand pose.

The following parameters are available in the “STEP 3: Target Annotation” module:

•  Input files: users can submit a UniProt ID that they are interested in. After clicking the “Find Target” button, a new window appears to show the four-character PDB IDs related to the UniProt ID. Users can also submit a four-character PDB ID in the step.
•  Target annotation: if the four-character PDB ID or UniProt ID can be found in our standardized and curated associations among drugs, genes, gene regulation, cancer outcome, cancer cell lines, and cancer types, a new table will be created to show the associations.
•  A PPI network: after clicking the “Target-related PPI” button, the screened targets can be projected to the PPI network constructed by AFFINOMICS and cancer and cardiac datasets in BioGRID and IntAct. Users can visualize and drag the nodes in the target-related PPI network using the mouse. Node colors represent the number of literature, and the darker the color, the lager the number of target-related literature. By placing the mouse on one node, users can observe the node-related representative literature.
•  Image download: TAIGET supports export of a high-quality picture of the PPI network, with the aim to facilitate academic research or education.

## Results and discussion

TAIGET consists of a docking service module, a target screen module, and a target annotation module, with the aim to facilitate traditional experiment researchers to identify potential targets. Compared with DrugComb (Zheng et al., 2021) that collected drugs, drug concentration, cell lines, drug response from drug combination screening studies, and monotherapy drug screening datasets, we text-mined and manually curated >14,000 PubMed literature works to construct associations among drugs, genes, gene regulation, cancer outcome, and cancer types for target annotation. Finally, 7,553 associations among drugs (6,109 types), genes (3,063 types), gene regulation, cancer outcome, cancer cell lines (2,170 types), and cancer types (73 types) were extracted from the literature. As we known, there is no active web server constructing such a simplified and comprehensive pipeline for target identification. In TAIGET, we only allow users to input ≤5 proteins at a time for docking because of the limitation of computing power. If users have more requirements, they could contact the authors by e-mails.

### Input files for TAIGET

For docking, users can input PDB IDs or UniProt IDs. For example, one user attempts to study the interactions of the serine/threonine kinase BRAF, a promising therapeutic target for lung cancer, with gefitinib, a tyrosine kinase inhibitor used as first-line therapy to treat non-small cell lung cancer. By providing UniProt ID P15056 of BRAF as the protein input, a new window will be created, showing 76 BRAF-related PDB files. Here, the user selects 1UWH as the protein structure, and meanwhile, provides the SMILE format of gefitinib COC1=C(C=C2C(=C1)N=CN=C2NC3=CC(=C(C=C3)F)Cl)OCCCN4CCOCC4 as the ligand input.

For the target screen, the user can input protein–ligand files created by oneself. TAIGET will identify interaction pairs and calculate interaction patterns for each interaction pair.

For target annotation, the user can enter a four-character PDB ID or a UniProt ID. For example, when the user provides 1UWH as an input, a new window will be created to show UniProt ID P15056. By clicking the UniProt ID with the mouse, a new table will be created, showing all the BRAF-related literature.

### Output files for TAIGET

In the docking step, a user can download ≤5 docked protein–ligand poses at a time or directly go to the next step. Here, we select AutoDock Vina for the docking of BRAF with gefitinib, which produces two poses.

In the target screen step, a table related to BRAF–gefitinib binding information will be created. The user can download the target-related information by clicking the “Download Results” button.

In the target annotation step, the user can get associations among drugs, genes, gene regulation, cancer outcome, cancer cell lines, and cancer types. Here, the user can provide 1UWH as an input to find three cancer-related literature works in a new table. For example, when the PubMed literature with doi of 10.2119/molmed.2011.00164 is identified, the user has access to the BRAF-related information, that is, sorafenib resulted in cell apoptosis of marrow stromal cells, nurse-like cells, and CLL cells by upregulation of BRAF and several related genes.

### Supported browsers and systems by TAIGET

The web server has been tested on all major browsers and operating systems (Table 2).

**TABLE 2:** Browser compatibility: TAIGET works in all major browsers and operating systems.

OS	Version	Chrome	Firefox	Microsoft Edge	Safari
Linux	Ubuntu 20.04.3 LTS	N/a	95.0	N/a	N/a
MacOS	OS X 10.11.6	95.0.4638.54	Not tested	N/a	11.1.2
Windows	10	96.0.4664.93	95.0	96.0.1054.53	N/a

## Conclusion

TAIGET combines docking and a BGMM-based target filter model to identify potential targets of small molecules, which is friendly to non-expert users via a GUI. More importantly, TAIGET involves a target annotation database, which contains curated associations among drugs, genes, gene regulation, cancer outcome, cancer cell lines, and cancer types derived from >14,000 PubMed literature works. This greatly favors experts and non-experts to explore target function and regulation in specific cancer cell lines.

## Data Availability

The original contributions presented in the study are included in the article/Supplementary Material; further inquiries can be directed to the corresponding authors.
